# Induced abortion, pregnancy loss and intimate partner violence in Tanzania: a population based study

**DOI:** 10.1186/1471-2393-12-12

**Published:** 2012-03-05

**Authors:** Heidi Stöckl, Veronique Filippi, Charlotte Watts, Jessie KK Mbwambo

**Affiliations:** 1 Department of Global Health and Development, London School of Hygiene and Tropical Medicine, Keppel Street, London, UK; 2 Department of Infectious Disease Epidemiology, London School of Hygiene and Tropical Medicine, Keppel Street, London, UK; 3 Department of Psychiatry and Mental Health, Muhimbili University College of Health Sciences, Dar es Salaam, Tanzania

## Abstract

**Background:**

Violence by an intimate partner is increasingly recognized as an important public and reproductive health issue. The aim of this study is to investigate the extent to which physical and/or sexual intimate partner violence is associated with induced abortion and pregnancy loss from other causes and to compare this with other, more commonly recognized explanatory factors.

**Methods:**

This study analyzes the data of the Tanzania section of the WHO Multi-Country Study on Women's Health and Domestic Violence, a large population-based cross-sectional survey of women of reproductive age in Dar es Salaam and Mbeya, Tanzania, conducted from 2001 to 2002. All women who answered positively to at least one of the questions about specific acts of physical or sexual violence committed by a partner towards her at any point in her life were considered to have experienced intimate partner violence. Associations between self reported induced abortion and pregnancy loss with intimate partner violence were analysed using multiple regression models.

**Results:**

Lifetime physical and/or sexual intimate partner violence was reported by 41% and 56% of ever partnered, ever pregnant women in Dar es Salaam and Mbeya respectively. Among the ever pregnant, ever partnered women, 23% experienced involuntary pregnancy loss, while 7% reported induced abortion. Even after adjusting for other explanatory factors, women who experienced intimate partner violence were 1.6 (95%CI: 1.06,1.60) times more likely to report an pregnancy loss and 1.9 (95%CI: 1.30,2.89) times more likely to report an induced abortion. Intimate partner violence had a stronger influence on induced abortion and pregnancy loss than women's age, socio-economic status, and number of live born children.

**Conclusions:**

Intimate partner violence is likely to be an important influence on levels of induced abortion and pregnancy loss in Tanzania. Preventing intimate partner violence may therefore be beneficial for maternal health and pregnancy outcomes.

## Background

Violence by an intimate partner is increasingly recognized as an important public and reproductive health issue. Intimate partner violence is highly prevalent, with population based surveys finding lifetime prevalence rates of physical and/or sexual partner violence between 15 and 71% [1]. As recognition of the prevalence of intimate partner violence and its negative sexual and reproductive health outcomes has grown [2,3], it is important to understand more about its association with induced abortion and pregnancy loss in different settings.

Studies conducted in low-income countries that used population-based, representative survey data, namely Bangladesh [4], India [5], Cambodia, the Dominican Republic, and Haiti [6] have found an association between physical and/or sexual intimate partner violence and induced abortion and pregnancy loss. The study conducted in Bangladesh, using the cross-sectional, nationally representative 2004 MEASURE Bangladesh Demographic Health Survey found that 76% of Bangladeshi women experienced violence from husbands and that those women who experienced violence from their husbands were more likely to report both unwanted pregnancy and a pregnancy loss in the form of miscarriage, induced abortion, or stillbirth [4]. The study from India drew on population-based data from two rural communities in Uttar Pradesh and Tamil Nadu collected in 1993 to 1994 and found support for an association between pregnancy loss and intimate partner violence [5]. In both of these studies the association even persisted after controlling for recognized explanatory factors such as education, poverty and parity. An analysis of the population based, representative Demographic and Health Survey data from Cambodia, the Dominican Republic and Haiti, conducted in 2000 and 2002 also showed an association between ever experiencing intimate partner violence and having had a pregnancy that ended in a non-live birth. Unfortunately this study did not differentiate between miscarriage, stillbirth and induced abortion [6].

Most studies from high income countries also support this association [7-9], for example a population-based, study from New Zealand, which had a similar design to the WHO multi-country study on which this paper is based on found that women who had ever experienced intimate partner violence were 1.4 times more likely to report they had ever had a miscarriage compared with women who had never experienced violence, and were 2.5 times more likely to report they had ever had an abortion, even after controlling for potential confounders [7]. Still, a prospective study among predominately low income, African-American pregnant women in the US did not find an association between intimate partner violence and pregnancy loss [10]. Until now, only one study using population-based data using the Cameroon Demographic Health Survey has been conducted in Sub-Saharan Africa to investigate whether exposure to physical, sexual and/or emotional intimate partner violence is associated with induced abortion and pregnancy loss [11]. This study found that women who were exposed to spousal violence were 50% more likely to experience at least one episode of pregnancy loss compared with women not exposed to abuse [11].

We hypothesized four pathways on how intimate partner violence can lead to adverse reproductive health outcomes in general and pregnancy loss in particular. The first pathway is direct, assuming that pregnancy loss is caused by direct physical trauma [12,13]. The other pathways are based on indirect associations. The first indirect pathway suggests that intimate partner violence is associated with pregnancy loss due to the women's stress related physiological responses to intimate partner violence, which can lead to low weight gain during pregnancy, restricted intrauterine growth, hypertension and infections during pregnancy [14,15]. The second indirect pathways is based on the assumption that intimate partner violence negatively impacts women's prenatal risk and health seeking behaviour, which may include alcohol and substance abuse during pregnancy as well as lower rates of antenatal care seeking and not seek hospital based delivery [2,16]. The third indirect pathway, which is also a potential explanation for the association between induced abortion, is that intimate partner violence can increase women's inability to negotiate contraceptive methods with her partner, which may lead to unintended pregnancy and thereby higher rates of induced abortions [16-18]. Even if unintended pregnancies are not terminated, evidence has shown an association with poor pregnancy outcomes [19]. Additional hypothesized pathways linking intimate partner violence and induced abortions are that abused women might be more likely to have had an induced abortion because their abusive partners forced them to have one or because the women feel unable to raise a child in an abusive relationship [7,20].

The WHO Multi-Country Study on Women's Health and Domestic Violence, on which this study is based found that the prevalence of physical and/or sexual intimate partner violence in Tanzania is high, with estimates of 41% in the Tanzanian capital Dar es Salaam and 56% in the rural region Mbeya [1]. A representative household survey of women aged 20 to 44 in the urban district of Moshi, Tanzania, conducted in 2002 to 2003 found a prevalence of 26% for experiencing threats of physical abuse, being subjected to physical abuse and forced intercourse by a partner [21]. In addition, rates of pregnancy loss and induced abortion are also expected to be above average in Tanzania, with the 2003 estimated global induced abortion rate being 29 per 1000 women aged 15 to 44, and the rate in Eastern Africa being 39 per 1000 women [22,23]. Given that induced abortions are illegal in Tanzania unless the woman's life is in danger, the majority of them are carried out under unsafe conditions and therefore carry a high risk of adverse maternal health outcomes [24]. In an ethnographic study in rural Mwanza, which drew on participant observation, group discussions and individual interviews, women revealed a variety of potential unsafe, clandestine methods to achieve illegal abortions. These include ingestion of dangerously high doses of medication or products not intended for human consumption, trusting people without appropriate skills, training or resources to perform manual abortions. Apart from the serious health consequences, this study also revealed that having to ask for an abortion made women vulnerable to financial and sexual exploitation due to the social ostracism they had to fear [24].

Due to the limited evidence on the association between intimate partner violence on induced abortion and pregnancy loss from population-based studies in sub-Saharan Africa, this study aims to investigate the relationship between intimate partner violence and induced abortion and pregnancy loss in the United Republic of Tanzania and to estimate its strengths relative to other explanatory factors, such as age, education, economic stress, marital status, extramarital affairs and number of children.

## Methods

This study analyses the cross-section data of the Tanzania section of the WHO Multi-Country Study on Women's Health and Domestic Violence, a large population based survey that gathered data from more than 24,000 women from a representatively selected samples of households in fifteen study sites in ten countries [1]. Conducted from 2001 to 2002, the Tanzanian section of the WHO multi-country study conducted representative surveys of women of reproductive age in the capital Dar es Salaam and Mbeya region. Dar es Salaam is made up of three municipals which are further subdivided into a total of ten divisions. These ten divisions are comprised of a total of 73 wards. Each ward in urban Dar es Salaam is further subdivided into streets, which itself are further subdivided into ten cell units. In rural Dar es Salaam wards are sub-divided into about 370 villages, which are also further subdivided into ten cell units. The later will have about 10-50 household. Mbeya region has six districts, each of which is further divided into divisions, which are then subdivided into wards. In the divisions Mbeya Urban and Mbeya Rural there are 53 wards, which further subdivide themselves. This administrative structure was used for the two stage cluster-sampling scheme. In Dar es Salaam all districts were included from which 22 wards from the list of 73 were selected, but for Mbeya, only two districts, urban and rural Mbeya, were included where 22 wards were selected from a list of 53. Using probability proportional to size of the wards (in each site) ten cell units were then selected randomly and their households were enumerated and mapped to allow random selection. Only one woman aged 15 to 49 was randomly selected per household and interviewed in complete privacy, except for infants younger than two years, to protect confidentiality and ensure safety [1]. After participation, all respondents received information about available local women's services, which also dealt with domestic violence. Women reporting thoughts on or attempts of suicide were seen by the supervisor responsible for the group and in a few cases referred to the psychiatrist responsible for the mental wellbeing of the team. No compensation was offered to participants.

The survey used female interviewers who were trained using a standardised three-week training course. The response rate at the household level was 100% in both sites and 96% in Dar es Salaam and 97% in Mbeya at the individual level [25].

The study adhered to the WHO ethical and safety recommendations for research on domestic violence against women [26] and received ethical approval from WHO Secretariat Committee for Research in Human Subjects and the research and publications committee of the then Muhimbili University College of Health and Allied Sciences. Regional and district directors and ward executive offices of the surveyed communities also gave their assent.

### Definitions of key measures used in analysis

The study adopted a number of working definitions. 'Ever partnered' included all women who report having ever been married to, ever lived with or currently have a steady, regular male sexual partner, regardless of the actual length of the relationship. 'Ever pregnant' included all women who reported having ever given birth to a child or having ever been pregnant.

In the survey women were asked directly whether they had experienced specific acts of violence. These include if her partner had slapped or had thrown something at her that could have hurt her, pushed or shoved her, hit her with a fist or with something else that could have hurt her, kicked, dragged, or beaten her up, choked or burnt her on purpose, threatened to use or actually used a gun, knife, or other weapon against her, physically forced her to have sexual intercourse when she did not want to, had sexual intercourse with her when she did not want to because she was afraid of what her partner might do, or forced her to do something sexual that she found degrading or humiliating. Additional questions about the frequency and timing of acts (ever, past year) were also asked. All women who answered positively to at least one of the questions about specific acts of physical or sexual violence committed by a partner towards her at any point in her life were considered to have experienced intimate partner violence.

Lifetime history of miscarriage, induced abortion, and stillbirth was assessed for all women through a single item asking if they ever had a pregnancy that ended in miscarriage, stillbirth or induced abortion. Recent research from Tanzania reported that surveys investigating 'miscarriage' and 'stillbirth' separately may not measure the intended outcome since these are Western concepts which do not directly translate into local categories [27]. Positive answers to these questions are therefore combined under the term pregnancy loss.

The additional explanatory factors in this analysis, as derived from prior studies on pregnancy loss and induced abortion [4,7,11], include location (urban/rural), age (continuous), education (no schooling/primary/secondary), partner's education (no schooling/primary/secondary), socio-economic status (low/medium/high), marital status (married/not married), partner having other wives or affairs (Yes/No) and number of live born children (continuous).

### Analyses

The analysis was restricted to ever partnered, ever pregnant women. To analyze the data, descriptive statistics were calculated and possible associations between factors were explored by conducting cross tabulations. Crude odds ratios (OR) between different forms of intimate partner violence and possible associations with induced abortion and pregnancy loss were estimated using bivariate logistic regression. Multivariate logistic regressions were used to obtain adjusted odds ratios (AORs), controlling for the more commonly recognized explanatory factors, such as women's and partner's age, education, socio-economic status, marital status and their partner having other wives or affairs, women's number of live born children and living in a rural versus urban area.

An odds ratio larger than one represents a greater likelihood of the outcome than for the reference category, and an odds ratio smaller than one represents a smaller likelihood compared with the reference category. A *p*-value below 0.05 was considered statistically significant.

Data were missing for less than five percent of respondents for most variables, and women with missing data were excluded from analyses with that variable. All analyses were conducted using STATA version 11, taking the cluster sampling design into account.

## Results

Of the 3270 women in the sample, 2712 (83.22%) had ever been in a relationship. Of these 2492 (91.89%) had ever been pregnant. Among all ever pregnant, ever partnered women, 49% (n = 1233) reported either physical or sexual violence by their partner, with 22.13% (n = 554) reporting only physical violence, 8.75% (n = 219) reporting only sexual violence and 18.38% (n = 460) reporting both. Having had a pregnancy loss was reported by 22.79% of ever partnered, ever pregnant women (n = 568, and 6.78% (n = 169) reported an induced abortion. Table 1 gives an overview of the demographic characteristics of women who experienced an induced abortion and pregnancy loss.

**Table 1:** Demographic characteristics and pregnancy loss and induced abortion of ever partnered, ever pregnant women in Tanzania (n = 2492)

	N	%	Pregnancy loss (n = 568, 23%)	Induced abortion (n = 169, 7%)
**Physical and/or sexual intimate** **partner violence ever**				

No	1259	50.52	46.48	39.05

Yes	1233	49.48	53.52	60.95

**Location**				

Dar es Salaam	1294	51.93	58.10	58.58

Mbeya	1198	48.07	41.90	41.42

**Woman's age**				

15-19	152	6.10	42.25	5.33

20-34	1544	61.96	55.99	49.70

35+	796	31.94	1.76	44.97

**Woman's education**				

Primary	1669	68.09	64.89	65.03

None	732	29.87	32.09	31.90

Secondary	50	1.6	3.11	3.07

**Socio-economic status**				

Low	1872	76.53	71.94	63.86

Medium	394	16.13	19.17	22.29

High	180	7.44	8.99	13.86

**Partner's age**				

15-19	6	0.24	68.96	0.61

20-34	992	40.02	31.04	31.52

35+	1481	59.74	0.00	67.88

**Partner's education**				

Primary	1796	74.21	69.29	72.22

None	403	16.65	20.11	14.20

Secondary	221	9.14	10.60	13.58

**Currently married**				

Yes	1455	58.39	61.44	50.30

No	1037	41.61	38.56	49.70

**Partner has another wife or affair**				

No	1115	47.27	40.99	44.16

Affair/Other wife	744	31.53	34.66	37.66

Don't know	500	21.20	24.45	18.18

**Number of live born children**				

< 3	1193	47.87	43.31	46.75

4 to 6	1082	43.42	46.30	43.79

> 6	217	8.71	10.39	9.47

As can be seen in Table 2, pregnancy loss was significantly associated with having experienced physical and/or sexual intimate partner violence or having experienced physical violence only but not sexual partner violence only or the experience of both physical and sexual partner violence. In contrast, induced abortion was significantly associated with having experienced sexual partner violence only and having experienced both physical and sexual partner violence, but not with physical partner violence only.

**Table 2:** Associations of lifetime experiences of intimate partner violence with induced abortion and pregnancy loss

	No violence	Physical IPV only versus no IPV^a, b^	Sexual IPV only versus no IPV	Both physical and sexual IPV versus no IPV
		**(22.13%)**	**(8.75%)**	**(18.38%)**

	%[95% CI]	%[95% CI]	%[95% CI]	%[95% CI]

**Pregnancy loss**	21 [18.7,23.5]	1.41** [1.12,1.78]	0.84 [0.58,1.22]	1.23 [0.94,1.61]

**Induced abortion**	5.20 [4.1,6.7]	1.29 [0.85,1.97]	2.12** [1.29,3.50]	1.86** [1.23,2.83]

***p < 0.001; **p < 0.01; *p < 0.05

^a ^IPV = Intimate partner violence

^b ^No IPV = No physical and no sexual intimate partner violence

The results of the binary and multivariate logistic regression analysis (Table 3) shows that that all explanatory factors, except women's education and marital status were significantly associated with pregnancy loss if examined on their own. Induced abortion was significantly associated with ever experiencing any form of intimate partner violence, women's and partner's age, socio-economic status, partner's education and marital status n the binary analysis. Once adjusting for the effect of other explanatory factors, only physical and/or sexual partner violence ever, women's age and number of live born children were the only factors that remained significantly associated with induced abortion and pregnancy loss. Women's socio-economic status was only significantly associated with induced abortion but not pregnancy loss, with rates of induced abortion increasing with socio-economic status.

**Table 3:** Crude and relative odds ratios (and 95% confidence Intervals) from binary and logistic regression analyses identifying factors associated with ever having experienced an induced abortion and pregnancy loss

	Pregnancy loss				Induced abortion			
	**Unadj. OR**	**[95% CI]**	**Adj OR**	**[95% CI]**	**Unadj. OR**	**[95% CI]**	**Adj OR**	**[95% CI]**

**Physical and/or sexual intimate partner violence ever**								

No	ref.		ref.		ref.		ref.	

Yes	1.23*	[1.02,1.49]	1.30*	[1.06,1.60]	1.65**	[1.18,2.31]	1.94**	[1.30,2.89]

**Location**								

Dar es Salaam	ref.		ref.		ref.		ref.	

Mbeya	0.72***	[0.60,0.88]	0.86	[0.69,1.08]	0.75	[0.54,1.04]	0.87	[0.60,1.27]

**Woman's age^a)^**	1.05***	[1.04,1.06]	1.06***	[1.03,1.08]	1.03**	[1.01,1.05]	1.05*	[1.01,1.09]

**Woman's education**								

Complete primary	ref.		ref.		ref.		ref.	

None	1.17	[0.95,1.44]	1.03	[0.80,1.32]	1.13	[0.81,1.57]	1.39	[0.96,2.01]

Complete secondary	1.83	[0.99,3.41]	1.35	[0.60,3.02]	1.64	[0.66,4.07]	1.34	[0.50,3.62]

**Socio-economic status**								

Low	ref.		ref.		ref.		ref.	

Middle	1.35**	[1.08,1.70]	1.18	[0.90,1.55]	1.73*	[1.13,2.64]	1.71*	[1.03,2.86]

High	1.42*	[1.01,1.98]	1.04	[0.66,1.64]	2.44***	[1.61,3.71]	2.06*	[1.12,3.81]

**Partner's age^a)^**	1.03***	[1.02,1.04]	1.01	[0.99,1.02]	1.02**	[1.01,1.04]	1.01	[0.98,1.04]

**Partner's education**								

Complete primary	ref.		ref.		ref.		ref.	

None	1.40**	[1.10,1.79]	1.17	[0.88,1.56]	0.87	[0.57,1.33]	0.74	[0.45,1.22]

Complete secondary	1.33	[0.98,1.80]	0.94	[0.64,1.38]	1.59*	[1.03,2.45]	0.76	[0.40,1.45]

**Currently married**								

Yes	ref.		ref.		ref.		ref.	

No	0.85	[0.69,1.04]	0.90	[0.72,1.14]	1.42*	[1.02,1.98]	1.31	[0.88,1.94]

**Partner has other wives or affairs**								

No	ref.		ref.		ref.		ref.	

Yes	1.35**	[1.08,1.69]	1.10	[0.86,1.40]	1.30	[0.92,1.85]	0.95	[0.64,1.41]

Don't know	1.45**	[1.12,1.88]	1.30	[0.99,1.72]	0.91	[0.59,1.41]	0.85	[0.53,1.34]

**Nr live born children^a)^**	1.04	[0.99,1.08]	0.88***	[0.82,0.95]	0.99	[0.92,1.07]	0.91*	[0.83,1.00]

***p < 0.001; **p < 0.01; *p < 0.05

^a) ^Measured as a continuous variable

## Discussion

Induced abortion and pregnancy loss is a common occurrence for women in this Tanzanian population, affecting almost one in four ever pregnant, ever partnered women. Induced abortion was reported by almost one in 14 of these women. This rates is comparably high compared to other African countries in the WHO multi-country study, for example, Ethiopia province were only up to two percent reported an induced abortion and Namibia where only up to 1.2% of women reported an induced abortion [24,15]. The finding also corresponds with Sedge et al's finding of high induced abortion rates in Eastern Africa [22]. Reports of intimate partner violence were also high, with 49% of ever pregnant, ever partnered women having reported physical or sexual violence by a partner. This rate is higher than those reported by the WHO multi-country study for Namibia (36%), urban and rural Thailand (41 and 47%) and urban and rural Brazil (29 and 37%) but lower than that found in rural Ethiopia (71%) [1]. The results of this study clearly show that physical and/or sexual intimate partner violence is a more important factor in understanding induced abortion and pregnancy loss than women's age, most socio-economic status variables and number of live born children. The significant association between induced abortion and sexual intimate partner violence, in conjunction with most induced abortions undertaken in Tanzania being illegal and therefore likely to be unsafe, suggests that sexual intimate partner violence may have serious reproductive health implications for women beyond significant psychological distress.

Several limitations of this study have to be acknowledged. First, it is based on cross-sectional data and therefore does not allow a sequencing of events to demonstrate a causal association. Second, this study has not set out to measure several factors that are also known to be associated with pregnancy loss, such as hypertension or infections during pregnancy nor the potential influence intimate partner violence might have on them. Third, intimate partner violence is a very sensitive issue and despite using highly trained interviewers, estimates are likely to be conservative. The same is likely to be true for measuring induced abortion and pregnancy loss, due to a general societal attitude that pregnancies should be concealed except from a few trusted individuals and that revealing a pregnancy loss carries social risks [27]. When interpreting the association between induced abortion and socio-economic status it has to be kept in mind that the difference between socio-economic status may occur either because of information bias, for example not understanding the question or not being willing to report or due to a real difference since women with a low socio-economic status might not able to access abortion services for financial and other reasons, such as less autonomy [28,29]. Other reasons for underreporting induced abortion are that abortion is punishable by seven years imprisonment in Tanzania, and fears of cultural disapproval and legal or religious sanctions [24]. Women might feel less safe to report an induced abortion. Overall, it may be more likely that women declare an induced abortion as a pregnancy loss, thereby underreporting the prevalence of induced abortion.

Nevertheless, this study adds to the growing evidence that intimate partner violence is strongly is associated with induced abortions and pregnancy loss. Previous research has suggested that the association is either direct or indirect. Pregnancy loss was seen as a direct consequence if the partner hit or kicked the woman into the abdomen during pregnancy [7]. Indirect explanations are that intimate partner violence may restrict women's autonomy or resources to care for themselves during pregnancy or that the trauma associated with their experiences of intimate partner violence makes them more vulnerable to current life stressors and that they therefore may have more reactive inflammatory responses [7]. A reverse explanation, given the unknown sequencing of events, is that violence by an intimate partner is triggered by the pregnancy loss, since childlessness is stigmatized in Tanzania and women may be blamed for poor reproductive health outcomes in Tanzania [27]. Induced abortion can therefore also lead to intimate partner violence if the partner did not want the woman to terminate a pregnancy [27]. A woman's decision to have an induced abortion may also be a consequence of intimate partner violence. For example, if abusive partners force women to terminate their pregnancy, women feel unable to raise a child in an abusive relationship [7] or if the partner forced the woman to become pregnant in the first place.

The association between sexual intimate partner violence and induced abortion established in this study lends support to recent studies which find that reproductive control is highly frequent in abusive relationships, with male partners threatening women to get pregnant, forcing women to unprotected sex, and sabotaging their contraception [20]. A representative household survey in Moshi found that 11% of women report their first sexual intercourse to be forced, with an additional 15% of women saying that it was unwanted [30]. This is concerning, given that other research from Tanzania also suggests that abortions seem to be the last resort for women with limited sexual negotiating power and low contraceptive options [24].

## Conclusions

This study provides previously unavailable population-level data on induced abortion and pregnancy loss and its association with intimate partner violence among representative samples of Tanzanian women. Its findings have implications for future research and service planning, since few studies and maternal health and family planning services currently consider intimate partner violence as an important contributing factor for pregnancy loss.

The findings suggest that preventing physical and sexual intimate partner violence has potential to improve maternal health and pregnancy outcomes. There are some promising models of intimate partner violence interventions focusing on antenatal care services [31]. A psycho-behavioral intervention based on the empowerment approach in Hong Kong was shown to significantly impact on both physical and sexual intimate partner violence and poor pregnancy outcomes, as well as improve women's mental health [32]. The applicability of this and other promising models in low income settings needs to be further explored and evaluated.

## Competing interests

The authors declare that they have no competing interests.

## Authors' contributions

HS carried out the statistical analysis for this paper and drafted the manuscript. VF provided support to the statistical analysis and helped draft the manuscript. CW and JKKM both participated in the study design and implementation. JKKM was the study coordinator in Tanzania. CW and JKKM reviewed a draft of the manuscript. All authors read and approved the final manuscript.

## Pre-publication history

The pre-publication history for this paper can be accessed here:

http://www.biomedcentral.com/1471-2393/12/12/prepub
